# Familial Amyloid Polyneuropathy treatment with Tafamidis – evaluation of one- and two-year treatment in Porto, Portugal

**DOI:** 10.1186/1750-1172-10-S1-O25

**Published:** 2015-11-02

**Authors:** Teresa Coelho, Ana Martins da Silva, Cristina Alves, Márcio Cardoso, Cecília Monteiro, Isabel Fonseca, Carla Rodrigues, Vanessa Costa

**Affiliations:** 1 Centro Hospitalar do Porto, Corino de Andrade Unit, 4099-001, Porto, Portugal

## Introduction

Transthyretin (TTR) related Familial Amyloid Polyneuropathy presents as a severe sensory, motor and autonomic neuropathy. Tafamidis, an oral drug that stabilizes TTR preventing amyloid deposition, was recently introduced in Europe to delay neuropathy progression in ambulatory patients.

## Objectives

To present Tafamidis efficacy and safety data after 12 and 24M treatment in patients from Porto, Portugal.

## Methods

Patients were evaluated at baseline, 6, 12 and 24M. Adverse events and body mass index were registered. Renal, thyroid, and liver functions were screened. Neuropathy impairment score (NIS), the Norfolk Quality of life (QoL) – diabetic neuropathy total score (Norfolk), this last only at baseline, 12 and 24M. Patients were classified as responders (NIS change across 12 and 24M<2) or non-responders (if greater).

Paired samples t test and ANOVA with repeated measures were used.

## Results

163 patients (92 males), with a mean age of 41.04 ± 11.68 years [26-80] and a mean duration of disease of 29.66 ± 17.48 months [4-90], completed a 12M evaluation. Body mass index remained stable throughout these 12M (3.13 vs. 3.14, p<0.008).

Mean NIS score decreased from baseline to 12M (2.35 vs. 2.34, p<0.694, ns) and Norfolk score improved between baseline and 12M (3.03 vs. 2.74, p<0.000).

Responders (n=112, 68,7%) showed a significant NIS-score decrease between baseline and 12M (2.24 vs. 2.05, p<0.000). Non-responders showed a significant increase across one year (2.56 vs. 2.88, p<0.000). Nonetheless, even in this group there was a Norfolk decreased in the same period (3.27 vs. 3.06, p<0.020).

The group that completed a 24M evaluation consisted of 104 patients (56 males), with a mean age of 40.04 ± 10.14 years [26-76] and a mean duration of disease of 32.03 ± 17.97 months [4-77]. Once again, body mass index remained stable throughout 24M (3.12 vs. 3.13, p<0.414, ns).

Mean NIS score increased from baseline to 24M (2.35 vs. 2.45, p<0.079, ns) and Norfolk score changed between baseline and 24M (3.10 vs. 2.85, p<0.001).

Responders (n=60, 57.7%) presented a significant NIS score decrease between baseline and 24M (2.13 vs. 1.97, p<0.002), while non-responders showed a significant increase across two years (2.63 vs. 3.04, p<0.000). On the other hand, non-responders' Norfolk decreased in the same timespan (3.22 vs. 3.06, p<0.029).

No safety problems were detected including, renal, thyroid and liver functions.

## Conclusion

Tafamidis stabilized 69% of patients treated for one year and 57% of patients treated for two years. Even patients classified as non-responders according to NIS score showed a good response both on QoL and BMI. No major safety issues were detected.

